# Comparison of collum femoris-preserving stems and ribbed stems in primary total hip arthroplasty

**DOI:** 10.1186/s13018-018-0981-0

**Published:** 2018-10-29

**Authors:** Mingqing Li, Can Xu, Jie Xie, Yihe Hu, Hua Liu

**Affiliations:** Department of Orthopedics, Xiangya Hospital, Central South University, Changsha, Hunan 410008 People’s Republic of China

**Keywords:** Total hip arthroplasty, Collum femoris preservation, Hip surgery, Implantation

## Abstract

**Background:**

This retrospective study investigated the relative benefits of using a collum femoris-preserving prosthesis or ribbed stem during total hip arthroplasty (THA).

**Methods:**

The clinical results were compared of patients who underwent THA, between January 2010 and December 2012, with either a CFP prosthesis or a ribbed stem (66 and 75 patients, respectively, aged 43.4 ± 10.8 and 42.3 ± 9.8 years). Patients were assessed using the Harris Hip Score (HHS), Western Ontario and McMaster University Osteoarthritis Index (WOMAC), 12-Item Short Form Health Survey (SF-12), and physical component summary (PCS) score. Intraoperative and postoperative complications and leg-length differences were noted.

**Results:**

The mean follow-up times of the CFP and ribbed groups were 67.2 ± 7.5 and 68.3 ± 7.2 months, respectively. The HHS, SF-12 MCS, SF-12 PCS, and WOMAC scores of the two groups were similar. The rates of periprosthetic femoral fractures and leg-length differences > 10 mm in the CFP group (10.6% and 13.6%, respectively) were significantly higher than those in the ribbed group (1.3% and 2.7%). The groups were similar regarding complications of osteolysis, ectopic ossification, dislocation, deep infection, deep venous thrombosis, thigh pain, and aseptic loosening. The survival rates of the CFP and ribbed groups were comparable (98.5% and 97.8%).

**Conclusion:**

The clinical results of the CFP and ribbed prostheses in young patients given THA were similar for Chinese patients. However, the CFP stem should be used with caution, given the high incidence of technical problems associated with implantation especially for Chinese patients.

## Background

Increasing numbers of patients are undergoing total hip arthroplasty (THA) because of highly successful rates and good clinical results [1]. The femoral neck is the most solid structure of the proximal femur and the center of stress distribution for the hip joint. Retention of the femoral neck preserves the trabecular systems of the metaphyseal cancellous bone, which allows a more even distribution of the physiological load along the diaphysis. Furthermore, protecting the blood supply to the femoral neck permits increased bone ingrowth [2, 3].

The collum femoris-preserving (CFP) short-stem prosthesis (Waldemar Link GmbH, Hamburg, Germany) is a cementless implant, especially appropriate for younger patients. Although there have been many reports of good early-to-midterm results with the CFP prosthesis [4–8], few studies have compared it with those that are more commonly used, in particular prostheses with a ribbed anatomical stem that require excision of the femoral neck for implantation [9, 10]. Burchard et al. [11] concluded from a virtual model that the CFP prosthesis has a stress-shielding effect and thus is harmful for bone remodeling. Thus, the question remains whether femoral neck-preserving hip prostheses perform better than traditional neck implants that require resection.

The present retrospective study compared the clinical results and complications of 66 patients who underwent THA with a CFP prosthesis, relative to 75 patients given a ribbed stem.

## Methods

### Patients and implants

Our Institutional Review Board approved this retrospective observational study.

The study comprised two cohorts. One cohort received a CFP short-stem prosthesis, and the other received a ribbed stem (both from Waldemar Link GmbH, Hamburg, Germany). Both stems are anatomical S-shaped stems; the principal difference being that the CFP short-stem prosthesis allows retention of the femoral neck. Patients were selected for either treatment by simple randomization.

From January 2010 to December 2012, 78 patients underwent THA by an experienced surgeon (the corresponding author) using the CFP stems. Eight patients with bilateral THAs were excluded from this study. An additional four patients were lost to follow-up. The remaining 66 were included in the study cohort.

The 66 patients who received CFP stems during THA were individually matched by age, gender, body mass index, and calendar year of the operation to 75 patients who underwent primary unilateral THA with ribbed stems (Table 1). The 75 patients were identified through the longitudinal registry as patients who underwent THA using ribbed stems. Thus, the patient demographics and clinical profiles of the two cohorts are similar.

**Table 1:** Demographics and preoperative diagnoses of patients undergoing primary THA

	CFP stems	Ribbed stems	*P* value
Total subjects, *n*	66	75	–
Gender, m/f	38/30	41/34	0.90
Age, years	43.4 ± 10.8	42.3 ± 9.8	0.52
Body mass index, kg/m^2^	25.3 ± 4.5	24.4 ± 3.9	0.20
Preoperative diagnosis			
Osteoarthritis	28 (42.4%)	60 (44.4%)	0.78
Avascular necrosis	30 (45.5%)	64 (47.4%)	0.79
Other	8 (12.1%)	11 (8.2%)	0.36
Follow-up, months	67.2 ± 7.5	68.3 ± 7.2	0.32

### Surgical data

All surgeries were performed by an experienced surgeon (the corresponding author) using a posterolateral approach after general anesthesia. All procedures were discussed by more than three experienced orthopedic surgeons and preoperatively planned according to the clinical manifestation and radiological inspections. A T.O.P. press-fit porous-coated TiAl6V4 acetabular cup (Waldemar Link GmbH) was used in both groups. Screws (Waldemar Link GmbH) were placed when the surgeon believed they would be helpful for acetabular fixation, based on the patient’s age and bone quality.

Postoperatively, all patients received an intravenous antibiotic to prevent postoperative infection. All patients received standard-of-care treatment. Low-molecular-weight heparin was given, and a lower-extremity venous pump was used for about 30 min, twice a day, to prevent thromboembolic incidents. Patients were instructed regarding hip exercises, and all therapy was supervised. Patients were mobilized as tolerated, starting with a walker and progressing to a cane. All patients were able to accomplish stair climbing before discharge from the hospital.

### Clinical evaluation

All patients agreed to enter the arthroplasty registry and were followed clinically and radiographically at 3 and 6 months postoperatively, and then annually. At each follow-up visit, patients completed the 12-Item Short Form Health Survey (SF-12) [12] (including mental and physical components) and the Western Ontario and McMaster University Osteoarthritis Index (WOMAC) [13, 14]. The evaluators also completed the Harris Hip Score (HHS) forms and documented the range of motion of the operative hip.

### Radiological evaluation

Standard anteroposterior radiographs of the pelvis and lateral radiographs of the hip, including the length of the total prosthesis, were obtained at regular follow-up intervals. Two qualified reviewers evaluated the radiographs for osteolysis and radiolucent lines > 1 mm using the zones of DeLee and Charnley [15] for the acetabulum and Gruen et al. [16] for the femur.

Complications, revisions, and implant failures were recorded. Leg length and offset was evaluated before and after surgery by tape measure [17, 18]. Offset was based on the contralateral hip; an offset difference ≤ 5 mm was considered a good result. For the purposes of this study, we compared the mean offset difference and percentage of hips with an offset difference ≤ 5 mm between the two cohorts. A tape was used to measure leg-length discrepancy, as the difference in lengths from the anterior spina to inner malleolus, between the operated and contralateral side.

Any patient who underwent a second surgery (i.e., femoral head or stem) was considered a survivorship failure.

### Statistical analysis

The HHS, WOMAC, and the Physical Composite Scale (PCS) and Mental Health Composite Scale (MCS) scores of the SF-12 were tested for improvement at the final follow-up, using an independent sample *t* test if the data had a normal distribution. A nonparametric test (Mann-Whitney *U* test) was used if the data had a skewed distribution. The chi-squared or Fisher’s exact test was used to determine if the incidence of complications was different between the two groups. A two-sided value of *P* < 0.05 was considered statistically significant. The statistical power of this study was 80%.

## Results

**Fig. 1:**
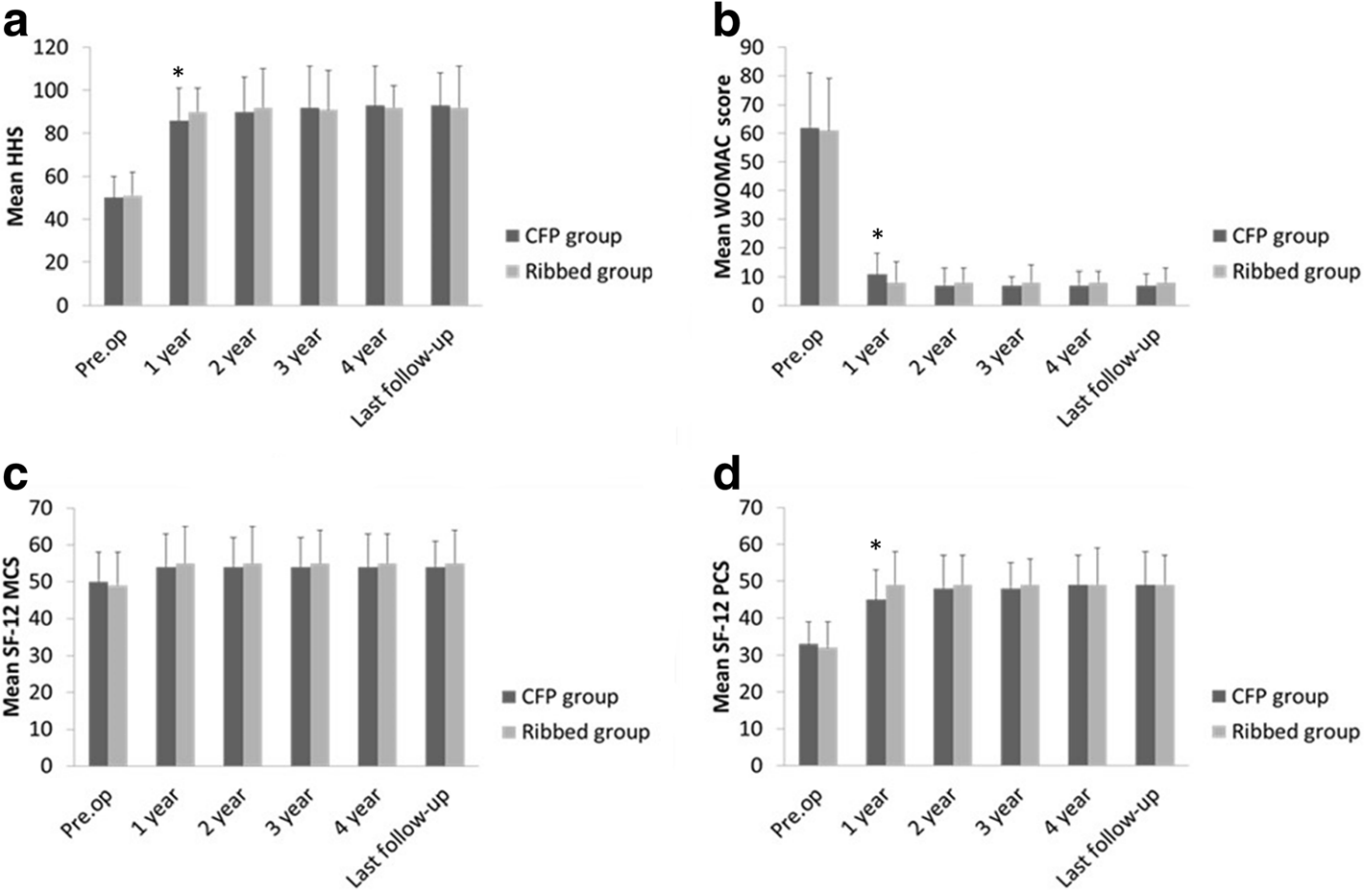
Histograms showing the mean HHS (**a**), WOMAC score (**b**), SF-12 MCS (**c**), and SF-12 PCS (**d**). The asterisk indicates a statistically significant difference between the CFP group and the ribbed group

Follow-ups of > 5 years were completed in both the CFP and ribbed stem groups. In both groups, at 1 year after THA and at each follow-up, the scores of the following were significantly improved relative to before surgery: HHS, WOMAC, SF-12 MCS, and SF-12 PCS (Table 2 and Fig. 1). At 1-year follow-up, the HHS, SF-12 MCS, and SF-12 PCS scores of the CFP group were significantly higher than that of the ribbed group. However, the mean WOMAC score of the CFP group was significantly less than that of the ribbed group (*P* = 0.01). At the other follow-up times, the scores of the two groups were comparable (HHS, WOMAC, SF-12 MCS, and SF-12 PCS).

**Table 2:** Results of HHS, WOMAC score, and SF-12 in the two groups

	HHS			WOMAC			SF-12 MCS			SF-12 PCS		
	CFP	Ribbed	*P*	CFP	Ribbed	*P*	CFP	Ribbed	*P*	CFP	Ribbed	*P*
Preoperative	50 ± 10	51 ± 11	0.53	62 ± 19	61 ± 18	0.72	50 ± 8	49 ± 9	0.44	33 ± 6	32 ± 7	0.32
1 year	86 ± 15	90 ± 11	0.03	11 ± 7	8 ± 7	0.01	54 ± 9	55 ± 10	0.49	45 ± 8	49 ± 9	0.00
2 year	90 ± 16	92 ± 18	0.44	7 ± 6	8 ± 5	0.21	54 ± 8	55 ± 10	0.48	48 ± 9	49 ± 8	0.43
3 year	92 ± 19	91 ± 18	0.72	7 ± 3	8 ± 6	0.20	54 ± 8	55 ± 9	0.44	48 ± 7	49 ± 7	0.34
4 year	93 ± 18	92 ± 10	0.61	7 ± 5	8 ± 4	0.13	54 ± 9	55 ± 8	0.43	49 ± 8	49 ± 10	1
Last follow-up	93 ± 15	92 ± 19	0.71	7 ± 4	8 ± 5	0.16	54 ± 7	55 ± 9	0.43	49 ± 9	49 ± 8	1

**Fig. 2:**
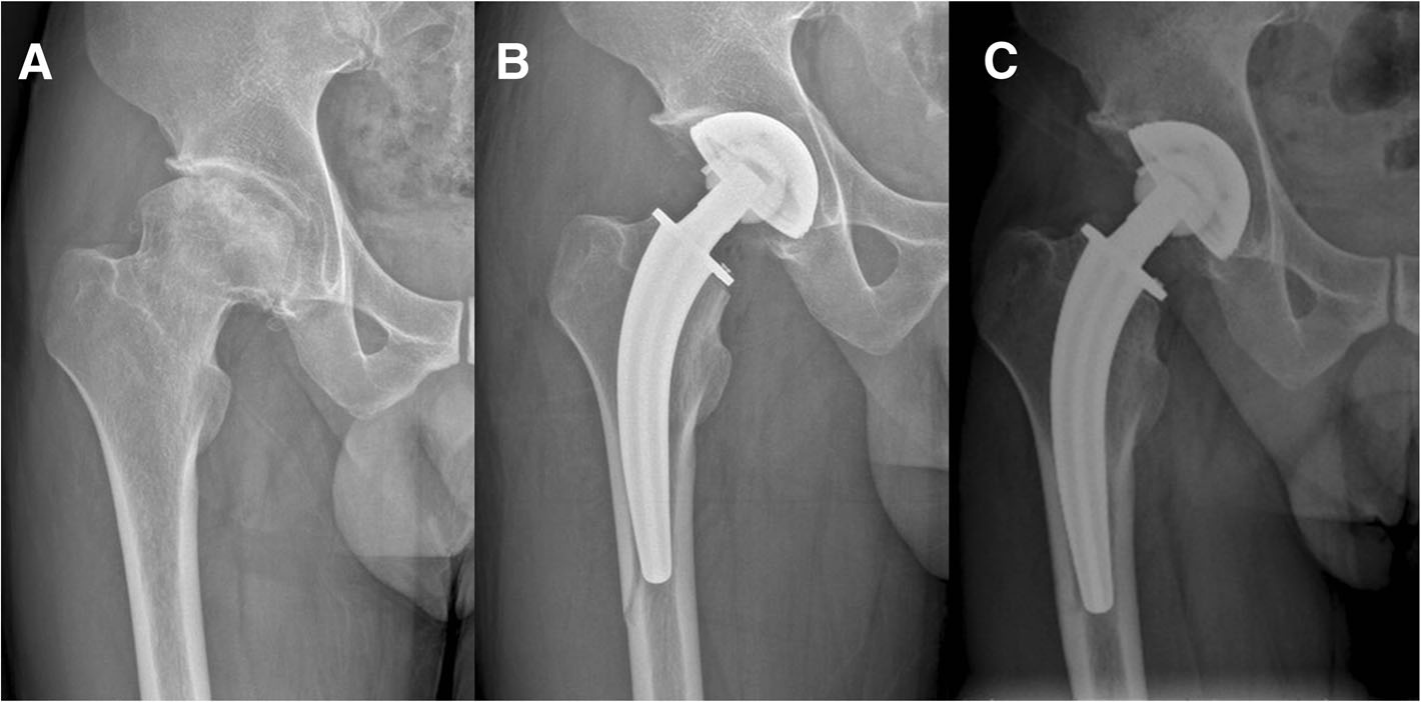
Preoperative anteroposterior radiograph of a 30-year-old man showing femoral head osteonecrosis of the right hip (**a**). Anteroposterior radiograph after THA using CFP stem showing PPF of the lateral femoral diaphysis (**b**). Anteroposterior radiograph at 1-year follow-up showing healing fracture (**c**)

In the CFP group, there were seven (10.6%) intraoperative fractures of the lateral femoral diaphysis at the tip of the stem, whereas only one (1.3%) occurred in the ribbed stem group (Fig. 2). No patient required additional treatment. At 5 days after surgery, all of the patients were allowed partial load-bearing (20 kg), and by 3 months, a gradual increase to full weight bearing. During this time, patients were asked to walk with the aid of crutches to assist with partial weight bearing. The fractures in all patients were healed at postoperative 8 months.

**Fig. 3:**
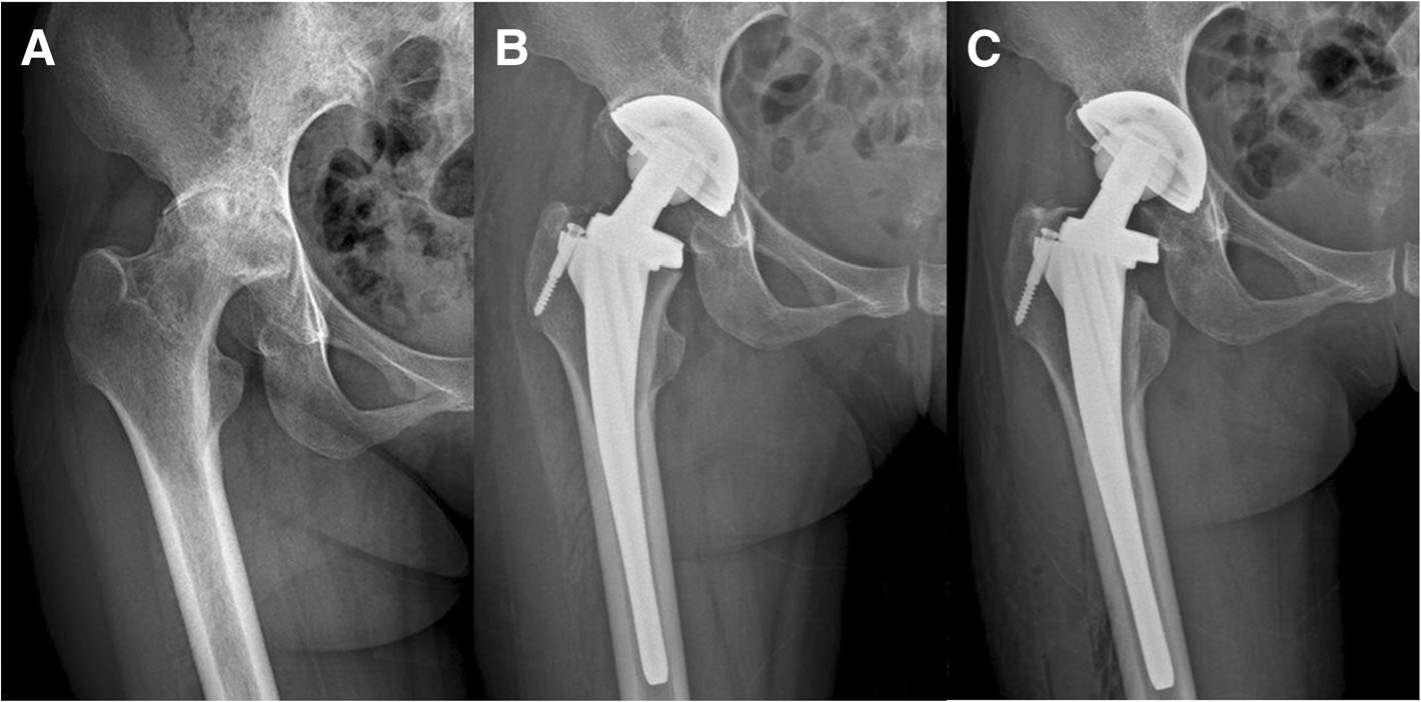
Preoperative anteroposterior radiograph of a 36-year-old woman showing femoral head osteonecrosis of the right hip (**a**). Anteroposterior radiograph 3 weeks after THA using a ribbed stem (**b**). Anteroposterior radiograph at 2-year follow-up showing osteolysis in Gruen zone 7 (**c**)

In the CFP group, radiological analysis showed that two (3.0%) patients developed periprosthetic osteolytic hips (both in Gruen zone 7). In the ribbed stem group, there were three (4%) osteolytic cases (one in Gruen zone 1, two in Gruen zone 7). A representative case is shown in Fig. 3. The difference in the incidence of osteolysis in the two groups was not statistically significant (*P* > 0.05).

Periarticular ossification was observed in seven hips: three (4.5%) in the CFP group and four (5.3%) in the ribbed stem group (*P* > 0.05). At the final follow-up, one (1.5%) hip dislocation had occurred in the CFP group (because of a tumble) and three (4%) had occurred in the ribbed group (two because of a tumble, the other because of acetabular cup malposition). The groups were statistically similar regarding rates of complications of deep infection, deep venous thrombosis, thigh pain, and aseptic loosening (all *P* > 0.05). Two hips required revision in the ribbed stem group, one because of recurrent dislocation, and the other because of deep infection; the latter was subsequently revised with two-stage reimplantation. Only one revision was performed in the CFP group (due to recurrent dislocation). The survival rate of the CFP group (98.5%) was similar to that of the ribbed group (97.3%; *P* > 0.05; Table 3).

**Table 3:** Intra- and postoperative complications of the CFP and ribbed stem patient groups, *n* (%)

	CFP	Ribbed	*P*
PPFF	7 (10.6%)	1 (1.3%)	0.026
Osteolysis or lucent lines	2 (3.0%)	3 (4%)	1.0
Ectopic ossification	3 (4.5%)	4 (5.3%)	1.0
Dislocation	1 (1.5%)	3 (4%)	0.623
Deep infection	0	1 (1.3%)	1.0
Deep venous thrombosis	0	2 (2.7%)	0.498
Thigh pain	0	1 (1.3%)	1.0
Aseptic loosening	0	0	–
Survival rate	98.5%	97.3%	1.0

*PPFF* periprosthetic femoral fracture

The results of leg-length differences are shown in Table 4. In the ribbed group, the majority of patients (65.3%) had equal leg length (≤ 5 mm), compared to 40.9% in the CFP group (*P* = 0.004). The percentage of patients with a leg-length difference > 10 mm in the CFP group (13.6%) was higher than that in the ribbed group (2.7%; *P* = 0.025).

**Table 4:** Limb length discrepancies of the CFP and ribbed stem patient groups

	CFP	Ribbed	*P*
0–5 mm	40.9% (27/66)	65.3% (49/75)	0.004
5–10 mm	45.5% (30/66)	32% (24/75)	0.101
> 10 mm	13.6% (9/66)	2.7% (2/75)	0.025

## Discussion

The CFP and ribbed stem are both anatomically S-shaped stems. The main difference between them is that the ribbed stem requires that femoral neck be cut, while the CFP does not. To investigate the relative benefits and suitability of the CFP and ribbed stem prostheses during THA for non-elderly patients, we retrospectively compared the clinical results of 66 patients given a CFP prosthesis during THA, with those of 75 patients given a ribbed stem. All the patients were followed for more than 5 years. The clinical and radiographic results suggest that both the CFP and ribbed prostheses perform well. We found that the use of the CFP stems did not improve the HHS, WOMAC, or SF-12 scores above that of the ribbed stems. On the contrary, use of the CFP stems was associated with an increase in the incidence of periprosthetic femoral fractures during surgery, and postoperative leg-length discrepancy, compared with the ribbed stems. Whether the CFP prosthesis is better than the ribbed stem for patients is still unknown, but the present study found that the CFP had no advantage over the ribbed stem.

Overall, the patients in the CFP group experienced seven intraoperative fractures of the lateral femoral diaphysis at the tip of the stem and six of these involved an exaggerated neck-shaft angle. The CFP prosthesis is designed in accordance with the normal anatomy of the femur, with two neck-shaft angles (126° and 117°); Jiang et al. [19] reported that the mean neck-shaft angle in a Chinese Han population was 133°. They also showed that adults younger than 60 years had a significantly higher neck-shaft angle. Hoaglund and Low [20] found the average neck-shaft angle in Hong Kong Chinese was 135°. The reasons for the differences in neck-shaft angle between these ethnicities are not known. All these results show that the average neck-shaft angle in Chinese people is larger than that in the CFP prosthesis. The compressive and tensile stresses surrounding the prosthesis are higher when the CFP prosthesis is used in patients with a smaller neck-shaft angle, and these stresses eventually lead to a fracture [21]. Thus, we concluded that the design of the CFP prosthesis may not be suitable for Chinese people.

The current study found that the incidence of intraoperative fractures with the CFP prosthesis was higher than that when the ribbed system is applied. There are few relevant articles regarding why intraoperative fractures occur when using either the CFP or ribbed stem prostheses, but such fractures were associated with the minimally invasive technique, press-fit cementless stems, female gender, metabolic bone disease, bone diseases that lead to altered morphology such as Paget’s disease, or intraoperative technical errors [22].

A difference in leg lengths is a well-recognized and common complication after THA [23, 24]. It is generally believed that differences of more than 1.5 cm can cause lower back pain, gait disorders, and general dissatisfaction [25]. However, it was determined that leg-length discrepancy was not associated with patients’ outcomes [25]. In the current study, the percentage of patients with a leg-length discrepancy of > 5 mm was higher in the CFP group than in the ribbed group. We consider that this is mainly because the operated femoral neck, with the length of the prosthesis neck in addition to the remaining patient’s neck, is longer than the patient’s original, or it can be due to the use of longer-neck femoral heads. However, other factors also influence leg-length discrepancy [26].

Radiographic evidence of osteolysis was detected in both the CFP and ribbed groups. Both the stems are collared prostheses, which can effectively prevent the subsidence of the stem, and may also lead to osteolysis in Gruen zone 7 due to stress shielding [27]. Another reason is the generation of wear particles [28], depending on implant design and manufacturing materials. Previous studies have shown that alumina-on-alumina articulations minimize osteolysis [29, 30].

The CFP prosthesis is designed especially for young (non-elderly) patients. According to the data we found, the CFP prosthesis failed to exhibit any advantages over the ribbed prosthesis. Whether the CFP prosthesis is more suitable for young patients is still unknown. Randomized controlled trials are warranted before the benefits of the CFP prosthesis are confirmed.

This study had several limitations. Firstly, the number of cases in the study was insufficient for good statistical power. Secondly, the study is retrospective, and all of the patients were from our hospital, constituting selection bias. Thirdly, we used only clinical measurements for the leg length, and these apparent assessments can differ from the more accurate radiographic evidence.

## Conclusions

The results indicate that both the CFP prosthesis and ribbed prosthesis significantly improved the preoperative HHS, WOMAC, and SF-12 PCS scores. However, differences between the two stems were insignificant, and the CFP prosthesis failed to exhibit any advantages over the ribbed prosthesis for Chinese patients. In addition, the CFP prosthesis is more prone to causing periprosthetic femoral fractures, so surgeons need to take preventive measures, especially for Chinese patients. Whether the clinical effect of the CFP prosthesis with femoral neck preservation is better than the ribbed stem with femoral neck truncation requires further study.

## Abbreviations

CFP: Collum femoris-preserving
HHS: Harris Hip Score
PCS: Physical component summary
SF-12: Item Short Form Health Survey
THA: Total hip arthroplasty
WOMAC: Western Ontario and McMaster University Osteoarthritis Index
